# City diplomacy as a strategic partnership: insights from Manchester and Wuhan

**DOI:** 10.1057/s41268-025-00365-6

**Published:** 2025-12-17

**Authors:** Weiwei Chen, Filippo Boni, Yameng Zhang

**Affiliations:** 1 The Open University, Milton Keynes, UK; 2 Xi’an Jiaotong-Liverpool University, Suzhou, China

**Keywords:** City diplomacy, Strategic partnerships, Non-material factors, Manchester–Wuhan relations, Subnational actors, Sustainable development

## Abstract

Cities are increasingly recognised as key agents in international relations, yet they are often overlooked as independent actors with their own priorities. By adapting the concept of strategic partnerships (SPs), originally devised for state actors, this article conceptualises city diplomacy as a distinctive, subnational SP. We argue that non-material factors—including mutual trust, shared objectives, and spatial synergies—are key in enabling effective cooperation among municipal actors. Drawing on 29 interviews conducted in the UK and China, participant observations, and policy documents, our investigation of the Manchester–Wuhan sister-city partnership, one of the longest-standing UK–China collaborations, illustrates how local partnerships can transcend national constraints to pursue shared priorities. By foregrounding these non-material yet pivotal drivers, our analysis reveals how subnational partnerships remain adaptable and resilient amid shifting geopolitical contexts. In doing so, we challenge state-centric assumptions in International Relations, highlighting the transformative potential of city diplomacy in addressing global development challenges. This research contributes to ongoing debates on the role of the nation-state in contemporary international relations by illustrating how city-level SPs can transcend national-level geopolitical tensions.

## Introduction

In June 2024, *The Economist* published a report titled *The Bright Side of China*, highlighting the rapid economic and social development of China’s “rising metropolises” (The Economist, 2024: 50). These so-called “second-tier cities” (including Wuhan) have consistently outperformed national averages in economic growth and urban population, reflecting a broader trend in which local governments are assuming prominent roles in regional and global affairs (Brenner, 2019; Curtis, 2021). One visible manifestation of this shift is China’s renewed focus on city diplomacy: since late 2023, Chinese municipalities have hosted visiting mayors from sister cities to revitalise cross-border collaboration, a testament to the growing agency of subnational actors within Beijing’s broader foreign policy (Voice of America, 2023).

This renewed prominence of city-level engagement aligns with a broader wave of interdisciplinary scholarship that reassesses the role of cities as global actors in both International Relations (IR) and development (Acuto, 2013; Brenner, 2019; Curtis, 2016, 2021; Madanipour, 2014). Yet, significant strands of the IR literature continue to treat cities primarily as geographic sites rather than as diplomatically proactive agents (Bouteligier, 2014; Ljungkvist, 2014; Acuto et al., 2017). Where cities do appear, they are often regarded as implements of national governments rather than distinctive subnational players capable of forging their own strategic partnerships (SPs). This state-centric framing typically foregrounds material dimensions such as trade volumes, foreign direct investment (FDI), or policy alignments, while overlooking non-material factors like trust, reciprocity, and spatial synergies.

However, as this article demonstrates, non-material forms of social capital^1^ can be decisive in initiating and sustaining subnational diplomatic ties. Drawing on the case of Manchester–Wuhan, this article shows how city-level cooperation has not only persisted but also expanded, even against the backdrop of deteriorating UK–China relations (BBC, 2022). By contrast, some partnerships—such as Manchester–St Petersburg and Arnhem–Wuhan—were dissolved or suspended in response to controversies and geopolitical pressures (*Clingendael *Institute, 2021; *The *Guardian, 2022), whereas the Duisburg–Wuhan partnership faced tensions yet remained active and relatively strong (Heberer and Shpakovskaya, 2022; Romano and Taube, 2024). We argue that Manchester–Wuhan’s durability stems from the embedded social capital that facilitates interactions even at times of tense relations at the national level.

Methodological nationalism (Wimmer and Glick Schiller, 2002) often obscures such subnational agencies, especially in relation to China (Mohan and Lampert, 2013; Schaefer et al., forthcoming). By attending to “soft” dimensions (i.e. personal relationships, cultural sensitivity, and community engagement) we can better explain how local stakeholders sidestep or mitigate macro-level geopolitical constraints. This perspective bridges “granular” and “grand” levels of analysis: while conventional IR analyses often focus on geoeconomic or state-to-state dynamics, city-level studies foreground how local actors craft and re-craft their partnerships from the bottom up (Acuto and Rayner, 2016). Put differently, subnational SPs do not merely execute top-down directives; they also co-create new initiatives, bringing together government, private sector, and community stakeholders under a defined, yet evolving, framework of cooperation.

Building on this perspective, the article addresses the central research question of how city-level SPs function as subnational partnerships and what factors allow them to endure, adapt, and produce tangible outcomes amidst shifting geopolitical conditions. To this end, the article makes three core contributions: first, it re-conceptualises city diplomacy as a subnational SP, emphasising how non-material factors such as multi-dimensional social capital can underpin sustained collaboration. In so doing, we speak to broader debates in IR and development, particularly those concerning multi-level governance (Hooghe and Marks, 2001) and the pursuit of global development goals (e.g., climate resilience, inclusive economic growth) by subnational entities (Parnell, 2016). Indeed, cities increasingly function as frontline implementers of the Sustainable Development Goals (SDGs), such as SDG 11 (Sustainable Cities and Communities) and SDG 13 (Climate Action) (United Nations 2015). By illustrating how Manchester–Wuhan navigated political frictions at the national level to cooperate on emerging fields like hydrogen energy, this study shows how city-based SPs can become incubators for tackling pressing global challenges, even when national-level relations are strained. Second, this article contributes to ongoing debates in international relations by analysing the growing significance of sub-national diplomacy. While recent scholarship suggests that nation-states have reasserted their authority in shaping city diplomacy according to national geopolitical agendas (Martinez & Bunnell, 2024), challenging the early 21st-century optimism about the autonomy of cities in global affairs (e.g., Barber, 2013), the Manchester-Wuhan partnership offers a counterpoint. It illustrates how inter-city social capital and trust-based networks can interact with, but also operate independently from, state-centred interests, thereby showing the relevance of sub-national actors in navigating geopolitical tensions. Third, the article offers an empirically grounded account (based on 29 original interviews plus participant observations) adapting Strüver’s (2017: 36) definition of SPs as “structured frameworks for collaboration between two or more parties to pursue shared interests and address common challenges” to the city scale, arguing that non-material forms of social capital are the decisive mechanism enabling sub-national sister-city SPs.

To develop these points, the article proceeds as follows. “City diplomacy in the IR literature” section outlines the conceptual framework by situating city diplomacy within IR scholarship on SPs, highlighting the often-neglected role of non-material factors in subnational arrangements. “Sister cities as strategic partnerships” section discusses data and methods, explaining the rationale for selecting the Manchester–Wuhan relationship. “Data and methods” section presents the empirical analysis, showing how specific intangible dimensions sustain an evolving city-level SP. Finally, the conclusion reflects on broader implications for IR and development, stressing the importance of dismantling methodological nationalism and recognising intangible but potent forms of social capital in city diplomacy.

## City diplomacy in the IR literature

In line with Van der Pluijm and Melissen’s widely used definition (2007: 11), we understand city diplomacy as “the institutions and processes by which cities, or local governments in general, engage in relations with actors on an international political stage with the aim of representing themselves and their interests to one another.” City diplomacy today encompasses a diverse range of practices through which municipalities engage in international relations, from participation in global governance forums to transnational advocacy and technical cooperation. Among these, city networks have emerged as a particularly dynamic form of inter-municipal collaboration (Taylor, 2011). Enabled by the power of networked governance and often supported by philanthropic actors, these networks have played a pivotal role in advancing global agendas such as climate action (Acuto et al., 2024), and in shaping the identity of cities as actors in global governance (Martinez and Bunnell, 2024). Within this evolving landscape, sister-city agreements, also referred to as twinning or international friendship city agreements, remain an important manifestation of city diplomacy. While the depth and impact of these agreements vary, they continue to offer a framework for long-term cooperation between cities, often encompassing areas such as economic development, cultural exchange, and education (Kihlgren Grandi, 2020).

While the roots of inter-city relations date back millennia, with examples ranging from Mesopotamian trade networks (Algaze, 2008) to cultural exchanges along the Silk Road (Hansen, 2012), modern city diplomacy gained prominence in the early 20th century. The founding of the International Union of Local Authorities in 1913 formalised city-level collaboration around issues like public health and urban governance. Later, in the 1980s and 1990s, globalisation and devolved governance further elevated the international role of cities. China’s Reform and Opening policy (1978) offers a vivid example: as Chinese municipalities gained autonomy, they significantly expanded their international partnerships beyond tourism and trade to include governance, environmental sustainability, and technology. By 2019, the number of sister-city agreements worldwide had surpassed 10,000, with China alone accounting for 2154 (Shpakovskaya, 2022: 37). Recent studies further highlight contemporary developments, including the growing strategic significance of EU–China inter-city relations (Kamiński et al., 2025) and the expanding scope of Chinese subnational diplomacy from a national–local perspective (Wei, 2021).

From an IR perspective, traditional schools of thought, particularly realism, have long framed cities as subordinate to national policies, implying minimal municipal autonomy in global affairs (Su and Chen, 2010; Xiong and Wang, 2013; Wang, 2022). Under realist assumptions, local actors merely implement directives from the central government without meaningful discretion. Such an approach faces difficulty explaining why or how certain municipalities independently pursue international initiatives, be it in climate adaptation, cultural exchange, or trade facilitation, that may deviate from state-level priorities. By contrast, liberal and multicentric frameworks (Rosenau, 1990; Duchacek, 1990; Curtis, 2021) describe a global order characterised by multiple centres of authority, where diverse non-state actors, including cities, participate in diplomacy. From this vantage point, cities engage in “para-diplomacy,” i.e., subnational cross-border actions to advance local interests in areas like environment, public health, or infrastructure (Ye, 2025; Li and Li, 2025). Constructivism also offers a valuable lens for understanding the evolving role of cities in global politics, particularly through the concept of governance. The latter has gained prominence in international relations as a framework that transcends the state-centric, Westphalian assumptions that dominated the field (Marchetti, 2021). Governance, in this context, is not confined to hierarchical state control but includes a multiplicity of actors and sites of authority. Constructivism, with its emphasis on the social construction of political realities, helps illuminate how cities emerge as meaningful actors in global affairs, not merely through formal governmental structures, but also via informal, networked, and often transnational forms of authority (Amen et al., 2011). Recent scholarship on Chinese para-diplomacy shows that subnational governments are not merely passive executors of state policy; they frequently pursue distinct local interests in international arenas (Ye, 2025). However, these studies also reveal that local autonomy varies widely, creating diverse patterns of city-level outreach (Li and Li, 2025).

In light of this heterogeneity, we argue that SPs, originally conceptualised for state-level interactions (Nadkarni, 2010; Wilkins, 2008, 2012; Strüver, 2017), can be adapted to subnational diplomacy to explain these dynamics. SPs typically involve structured, yet flexible collaborations aimed at shared interests. Applied to cities, the SPs framework not only elucidates the formal dimensions of cooperation (for instance, sister-city agreements) but also highlights the non-material factors—such as trust, cultural alignment, and people-to-people ties—that shape subnational internationalism (Boni, 2023). These non-material factors enable cities to select their international partners, decide which priorities (e.g., economic development or social welfare) to pursue, and continually adjust their joint initiatives in response to evolving local or global conditions.

### Sister cities as strategic partnerships

As previously noted, we argue that sister-city agreements can be conceptualised as SPs. Nadkarni (2010: 46–48) defines SPs as “diplomatic instruments that allow for hedging against all eventualities while allowing for the common pursuit of mutual interests.” Strüver (2017: 36) refines this by emphasising SPs as “structured frameworks for collaboration between two or more parties organised in a loose and non-binding way, aiming to pursue shared interests and address common challenges across various issue areas.” Building on Wilkins (2008, 2012), Parmeswaran (2014), and Boni (2023), it is apparent that sister-city SPs share several key features that mirror these more conventional SPs.

First, there is a need for a structured framework of collaboration. This is normally institutionalised and, in the case of sister cities, involves a signing ceremony between the two Mayors to establish the importance of the partnership. The various institutional configurations vary depending on the goals and actors involved, but the signing agreements normally identify the areas of cooperation, the main actors involved, and the mechanisms created to advance collaboration. Second, strategic partnerships have very high levels of flexibility. As previously noted, they are designed to represent broad platforms that embrace a number of areas for cooperation, which can change and evolve according to the needs of the moment. Items of cooperation can be added or removed flexibly, which makes these mechanisms particularly adaptable across time. Third, partnerships are the manifestation of the participating actors’ willingness to commonly pursue joint interests and mutual goals while leaving aside more conflictive issues. This goal-oriented nature is an important feature that, as we will see in the empirical parts of the paper, ensures the partnership’s continuity. Fourth, the aim of the partnership is often to develop a platform for cooperation for the sake of cooperation. A key aspect in this regard is their ability to reduce uncertainties, not only vis-à-vis the international environment but also among partners (Strüver, 2017). They have the potential to reduce uncertainty through repeated exchanges, to foster and deepen trust, and thus to facilitate future cooperation. Fifth, as recently introduced by Boni (2023), it is also important to account for a non-material dimension within this framework, as it helps recalibrating existing understandings of how bilateral interactions at the sub-national level operate. This is because focusing solely on the formal arrangements or material aspects (e.g. trade and investments) characterising city diplomacy risks overlooking the equally critical informal and non-material interactions. City diplomacy engages in people-to-people interactions often focused on cultural understanding, social exchange, and economic collaboration (Zhang et al., 2021), and it is therefore critical to incorporate these aspects into our analysis of how city diplomacy operates.

By interpreting sister-city agreements through the lens of SPs, we establish a conceptual basis for examining how local actors establish and sustain bilateral ties, why these arrangements can adapt over time, and what conditions allow them to transcend or, in some cases, succumb to geopolitical pressures. This perspective points us toward the specific mechanisms by which city partnerships function, the flexibility they exhibit in responding to shifting national or global priorities, and the broad scope of collaborative activities they can undertake. In the ensuing sections, we use this SPs framework to dissect how Manchester and Wuhan have built, maintained, and reconfigured their cooperation across multiple domains. Unlike state‑level SPs, which are typically formalised, security‑sensitive, and tightly coupled to national agendas, city‑level SPs are looser platforms anchored in trust, shared objectives, and spatial synergies, enabling rapid pivoting (e.g., to hydrogen collaboration) when national politics tighten.

## Data and methods

To contextualise the Manchester–Wuhan partnership, we conducted an extensive review of secondary sources in both Chinese and English on UK–China sister-city relationships (see Table 1). This included analysing a dataset of approximately 100 to 110 active or recent pairings, encompassing formal sister-city agreements, friendly exchange relationships, and other types of formalised partnerships. Among these, about 25 to 30 partnerships were established over three decades ago (before 1993). Examples include Manchester–Wuhan (1986), Taizhou–Newcastle (1985), and Xiamen–Cardiff (1983).^2^

**Table 1:** Distribution of UK–China sister-city partnerships by depth and longevity

Category	Num (approx.)	Notes
Total number of active or recent pairings	100–110	Includes formal agreements, friendly exchange relationships, and other formalised partnerships
Partnerships over three decades	25–30	Established in 1993 or earlier (e.g., Wuhan-Manchester, Xiamen-Cardiff)
Diverse & deep collaboration partnerships	15–20	Multi-sector engagement sustained over time (e.g., Manchester-Wuhan, Hangzhou-Leeds)
Primarily symbolic partnerships	40–50	Limited or sporadic interaction, often ceremonial or policy-oriented with minimal joint projects

Source: Authors’ compilation and analysis based on various sources, including China Daily (2024), House of Commons Library (2024), Manchester City Council Archives (2020–2022), MOFCOM (2022), Man and Chan (2021), LGA (2021), UK Government Office for Science (2016a, 2016b), and Embassy of the People’s Republic of China in the United Kingdom (2010)

The dataset categorises these partnerships according to their depth and longevity (see Table 1). While some city pairings demonstrate substantial engagement, with cooperation spanning trade, education, technology, and cultural sectors, many remain largely symbolic. Approximately 15 to 20 partnerships exhibit sustained multi-sector collaboration, including Chengdu–Sheffield, Hangzhou–Leeds, and Manchester–Wuhan. In contrast, 40 to 50 pairings maintain predominantly symbolic ties characterised by limited or sporadic interactions, often restricted to ceremonial visits or policy statements without substantive cooperation.

As demonstrated by Table 1, longevity alone does not guarantee substantial cooperation. Some partnerships initially pursued ambitious goals but became symbolic, whereas others evolved into concrete collaborations, highlighting the importance of accounting for multiple influencing factors.

We selected the sister-city relationship between Manchester and Wuhan as a “typical case” (Seawright & Gerring, 2008; Zhang et al., 2025) to investigate how city diplomacy can evolve over multiple decades and engage diverse stakeholders across various sectors. Formally established in 1986, the Manchester–Wuhan partnership has grown significantly since 2003, encompassing collaborations in trade, technology, education, sports, culture, arts, and climate initiatives—areas commonly featured in sister-city arrangements worldwide (Zelinsky, 1991; Cremer et al., 2001). As references in local media and municipal archives show, the Manchester–Wuhan ties have not only persisted but have adapted to political sensitivities, including shifting UK–China relations (BBC, 2022). By contrast, partnerships such as Manchester–St Petersburg and Arnhem–Wuhan were terminated or suspended in response to geopolitical and rights-related controversies, whereas Duisburg–Wuhan experienced strain yet remained active (Heberer and Shpakovskaya, 2022; Romano and Taube, 2024; C*lingendael *Institute, 2021; *The *Guardian, 2022). We argue that the durability of the Manchester–Wuhan partnership stems from embedded social capital, which facilitates flexible problem-solving and helps maintain trust despite broader national tensions.

Primary data collection relied mainly on semi-structured interviews and participant observations. Between October 2022 and June 2024, we conducted in total 29 semi-structured interviews—27 in the UK (mostly in Manchester) and two remotely with Wuhan officials via WeChat. A consistent interview guide was used for all interviews, with slight adaptations according to stakeholder groups (see Appendix 1 for detailed interview questions). Stakeholders included government officials at both city and regional levels, representatives from investment promotion agencies (both Chinese national and local, as well as those based in Manchester), business leaders (Chinese and British), university representatives, research and training institution members, and participants from the Chinese community, including student representatives. This broad stakeholder engagement provided comprehensive insights into the Manchester–Wuhan partnership dynamics.

Practical constraints such as geopolitical sensitivities and COVID-19 restrictions limited direct interviews in China, slightly constraining the depth of Wuhan-based perspectives. However, these limitations were mitigated by comprehensive secondary data analysis and local participant observation, ensuring rigorous methodological triangulation.

To ensure participants could speak openly, confidentiality was assured unless explicit consent for identification was given. Interviews were coded systematically using identifiers based on nationality, sector, and a unique number for each interview (e.g., BC1 for the first British corporate interview). The following codes were used: BC = British Corporate; BG = British Government; CG = Chinese Government; BI = British Institute; CI = Chinese Institute. Participant observation, coded as “PO” with unique identifiers (e.g., PO1), provided additional insights into key events, including: (1) a virtual meeting between Manchester and Duisburg officials (PO1); (2) Manchester China Institute-led events (PO2–PO4), such as the International Photo Competition and Manchester China Friendship Programme; and (3) a focused group discussion with Chinese student representatives (PO5). The data were analysed using thematic coding in NVivo 14, which enabled systematic categorisation of key themes and patterns.

Data analysis utilised thematic coding via NVivo-14 software, employing a structured, two-stage thematic analysis. Initially, five themes—structured collaboration, flexibility, goal orientation, long-term orientation, and trust networks—were deductively derived from strategic partnership literature (Strüver, 2017; Wilkins, 2008; Nadkarni, 2010). Subsequently, iterative inductive coding of interview transcripts and archival data refined these themes, aligning closely with empirical findings. Regular cross-checking by two independent coders ensured reliability and consistency. This produced four analytical themes tailored specifically to the Manchester–Wuhan partnership (see Table 2).

**Table 2:** Analytical themes

Analytical theme	Definition	Source
Structured collaboration	Institutional yet adaptable frameworks for formal/informal interactions	Deductive
Flexibility	Dynamic ability to shift towards new areas or projects (e.g., hydrogen energy)	Inductive refinement
Goal orientation	Clear objectives targeting outcomes (e.g., environment, cultural inclusivity)	Deductive/Inductive
Long-term collaboration platforms	Enduring institutional pillars—such as higher‑education programmes and long‑term business partnerships—that anchor multi‑decade cooperation and create cumulative value	Inductive

Source: Authors’ compilation from primary interview data.

This structured analytical approach enhances methodological clarity, explicitly bridging empirical insights with theoretical frameworks from the strategic partnerships literature (Strüver, 2017; Nadkarni, 2010). The subsequent empirical analysis (Section 4) explores how non-material factors, particularly varied forms of social capital, enable resilience and adaptability within city diplomacy. By demonstrating how these intangible factors operate in the Manchester–Wuhan partnership, our analysis highlights the potential of subnational strategic partnerships for sustaining innovative collaboration despite national-level geopolitical pressures.

## The Manchester–Wuhan sister-city partnership: an empirical analysis

In this section, we examine the Manchester–Wuhan partnership through a city-diplomacy-as-SP framework, focusing on how non-material aspects enable the relationship to evolve, adapt, and yield concrete outcomes. The analysis is organised around four key themes—structured collaboration, flexibility, goal orientation, and long-term orientation—reflecting the multidimensional nature of subnational SPs.

### Structured collaboration

Sister-city agreements between Manchester and Wuhan date back to the mid-1980s and initially provided a formal structure for collaboration on education, culture, and trade, as the basis for the partnership’s early engagements. However, our analysis indicates that this formal structure alone was not sufficient. Instead, a gradual process unfolded, where official agreements were significantly strengthened by interpersonal relationships and a shared vision of city-to-city collaboration.

The Tiananmen Square incident in 1989 exemplifies how these non-material aspects helped sustain the partnership during periods of heightened political sensitivity. While many Western cities, including some in the UK, paused or terminated their ties with Chinese counterparts during this period, Manchester took a more cautious and consultative approach. City officials first engaged local Chinese representatives before issuing any public statements, seeking input from local Chinese community members to balance major concerns with the desire to maintain dialogue (BG1-a). A former Manchester City Council (MCC) leader reflected, “We’re true friends, but real friends also express concerns about actions they dislike. This trust allowed us to maintain the partnership while navigating political tensions” (BG1-a). This statement functions analytically as evidence of trust: interpersonal rapport sustained cooperation while lowering the political costs of continued engagement. Rather than completely terminating ties, Manchester temporarily reduced formal interactions, enabling a relatively smooth re-engagement in subsequent years. Current policymakers in Greater Manchester continue to regard this event as a precedent for tackling politically sensitive matters, affirming that thoughtful engagement can uphold ethical stances while preserving future opportunities for subnational cooperation (BG1-b, BG2-a).

Manchester’s Chinese community^3^ played a similarly pivotal role in shaping the early years of the partnership. Drawing on historical ties around trade, migration, and local industry, members of this community championed Wuhan as a sister-city partner, aligning official processes with local realities (BG1-a, BG1-c). Influential figures such as “Uncle Charlie”^4^ bridged institutional negotiations with informal trust networks, ensuring early stakeholder buy-in (BI6-d). Over time, this “grassroots diplomacy” found resonance in both cities, facilitating the expansion of bilateral programmes, indicating community brokerage to pursue shared objectives.

Another key and more recent example is the establishment of the Manchester China Forum (MCF), a public-private partnership established in 2013 as a special purpose vehicle (SPV) to strengthen ties between Greater Manchester and Greater China (MIDAS n.d.). By coordinating actors from governance, higher education, and the private sector, the MCF helped transform the sister-city relationship into a dynamic platform. As a former MCC leader noted, “We didn’t just rely on formal agreements; we always kept the personal connections alive, which were crucial for adapting to new contexts and challenges” (BG1-b). She further recalled that the MCF’s three-pillar mechanism—governmental collaboration, institutional partnerships, and people-to-people exchanges—had been informally in place for years, illustrating how sustained personal networks can pre-empt and then bolster institutional structures (BG1-c). These dynamics display two important points: first, the importance of interpersonal ties in the evolution and adaptation of sister city partnerships; second, the relevance of spatial synergies, whereby networked institutions anchored cooperation across sites and sectors.

Some observers might argue that Manchester’s posture merely followed UK national directives at the time or reflected pure economic self-interest. Indeed, interviews confirm that national policy and trade considerations did play supportive roles (BG2-a, BC1). However, city officials and private-sector respondents consistently emphasised longstanding personal rapport and community-level connections as the key to sustaining cooperation amid political headwinds. As one MCC official remarked, “Economics alone wouldn’t have carried us through that tense period; it was the trust networks we’d built that made the difference” (BG1-b). Consequently, macro-level policy directives or cost–benefit calculations—though relevant—do not fully explain the long-term resilience of this subnational collaboration.

Taken as a whole, the Manchester–Wuhan story demonstrates how formal institutional frameworks can be translated into adaptive, trust-based partnerships that evolve to meet both local and international demands. The partnership begins with official charters, continues through frequent interpersonal exchanges that cultivate trust, and is then deployed through concrete initiatives with broader institutional and developmental relevance, thereby illustrating how subnational SPs can transcend purely symbolic gestures or state-centric constraints. The partnership’s capacity to manage political sensitivities, mobilise community stakeholders, and steer innovative programmes speaks to the central role of non-material aspects in city diplomacy.

### Flexibility: adapting to shifting contexts

An interviewee from the Manchester China Institute (MCI), a Manchester-based academic research institute, described the Manchester–Wuhan relationship as “much looser and more flexible” than rigid national-level directives, explaining that “it’s more like, ‘Here’s an opportunity, let’s take it if we can’” (BI2-a). This flexibility is a crucial characteristic of city diplomacy, where formal structures exist but local actors can quickly respond to new opportunities or challenges, often bypassing geopolitical tensions. Hydrogen energy exemplifies this dynamic, emerging in the early 2020s—amid a period of heightened political caution in UK–China relations—as a mutually beneficial focus that aligned with sustainability goals and provided a “safer” domain for bilateral engagement. An interviewee at the MCF observed that hydrogen energy “represented a strategic area where both cities could collaborate without fear of political backlash,” thereby offering “a less contentious platform for cooperation” (BI1-b).

Crucial to advancing such initiatives was the role of the UK National Committee on China (UKNCC)^5^—a Manchester-based NGO that convened relevant government agencies, research institutions, and private firms from both sides (BI6-a; BI6-d). By hosting the China–UK Hydrogen Energy Cooperation Forum, the UKNCC effectively bridged political and organisational divides, introducing influential Chinese partners (such as the China Hydrogen Energy Special Committee and the State Power Investment Hydrogen Energy Company) to Manchester’s existing infrastructure for research and innovation (Palcan, 2022) and accelerated the actual implementation. One interviewee stressed that “without platforms like the Forum and the networks established by the UKNCC, it would have been challenging to keep technical and policy discussions on track amid political uncertainties” (BI6-a).

Economic or policy incentives alone did not entirely explain how collaborative efforts persisted during a period of political tensions in UK–China relations, characterised by the enactment of the Hong Kong National Security Law (BBC, 2020a), the UK’s decision to exclude Huawei from its 5G networks (BBC, 2020b), and renewed policy debates on “de-risking” Chinese investment (European Commission, 2023). Interviews consistently pointed to non-material factors, especially long-standing personal networks and a tradition of cross-cultural engagement that helped carry projects through. A leader from Manchester’s Chinese community remarked, “We have the quality of connections and relationships that kept projects alive, even when the bigger picture was turbulent” (BI6-c). Rather than passively following national policy shifts or simply chasing commercial benefit, participants drew on trusted ties to identify newer, lower-risk fields of cooperation.

Spatial synergies also underpinned this flexibility. Manchester’s expertise in green technology complemented Wuhan’s industrial strengths, creating what one MCF representative called “a synergy that met the sustainability goals of both cities” (BI6-d). Beyond short-term outputs, the emphasis on hydrogen energy signalled a shared cognitive stance toward environmental issues, a convergence that resonates with broader global development agendas—such as climate action and inclusive innovation—that city-level actors increasingly champion (Curtis, 2021).

The capacity of city partnerships to adapt quickly and exploit new opportunities highlights flexibility as a core element of subnational SPs. While formal frameworks provide essential parameters, the Manchester–Wuhan example demonstrates that local agency, grassroots diplomacy (i.e. community-driven diplomacy), and the alignment of local strengths are critical for sustaining cooperation. Even amid geopolitical tensions, city partnerships can shift focus to new projects—such as hydrogen energy—that meet both local needs and broader global challenges.

### Goal-oriented collaboration: advancing sustainability and inclusivity

The Manchester–Wuhan partnership also demonstrates a goal-oriented dimension, focusing on specific initiatives designed to address shared challenges and improve local communities. In contrast to purely symbolic ties, these initiatives show how clearly defined objectives—ranging from environmental sustainability to cultural inclusivity—translate subnational diplomacy into tangible, long-term impacts. While economic or policy interests could explain some aspects of these outcomes, interviews and documents reveal a deeper reliance on non-material aspects such as relational trust, shared objectives, and local empowerment. By aligning on common goals, both cities not only address local needs but also contribute to broader international relations and development agendas such as climate action, resilience-building, and social cohesion.

#### Environmental sustainability: co-developing urban resilience

A key illustration of this goal-oriented nature lies in their collaboration on environmental sustainability, a critical concern rooted in both cities’ industrial legacies. Wuhan, historically known as “the city of one hundred lakes” (Guardian, 2019), faces escalating flood risks due to rapid urbanisation and reduced natural water absorption capacities (*Hellofuture*, 2021). Similarly, Manchester has confronted its own environmental challenges, including the aftermath of industrial pollution in its waterways (ibid). These parallel challenges provided a catalyst for knowledge exchange, leading both cities to leverage each other’s innovations and experiences in green infrastructure.

Wuhan’s Sponge City initiative^6^—designed to enhance urban resilience by improving water absorption and purification—exemplifies how a locally devised strategy can form the basis for international collaboration. When Manchester launched its West Gorton Park initiative—an urban green space integrating stormwater management, biodiversity enrichment, and recreational features—it drew inspiration from Wuhan’s model. As one MCC policymaker explained, “This project not only underscores our commitment to sustainable development but also illustrates the practical benefits of learning from Wuhan’s approach” (BG1-b). Beyond infrastructural similarities, the alignment extended to spatial synergies—Manchester’s research-driven design expertise complemented well with Wuhan’s industrial capacity, allowing each city to co-develop tailored solutions that matched their respective urban-development goals.

Moreover, the collaboration extended beyond infrastructure projects. A senior Wuhan municipal official recounted how Manchester’s post-industrial transformation shaped Wuhan’s urban tourism strategies (CG2). In 2006, he participated in a six-month training programme hosted by one of Manchester’s academic institutes, where he was embedded in the municipal governance structure to learn firsthand from local officials. During this experience, he studied Manchester’s approach to converting former factories into cultural and commercial hubs, knowledge that later drove significant regeneration projects upon his return. As he remarked, “We didn’t simply deindustrialise; we upgraded and transformed, which became a guiding principle for Wuhan’s long-term development strategy” (CG2). Participants described the personal connections forged during the programme as a “valuable by-product” that sustained dialogue well beyond the training itself. This sequence of identified urban challenges, targeted knowledge exchange, and trust-building interactions ultimately led to tangible policy transformations in Wuhan.

While critics might assume economic self-interest or central directives to be sufficient explanations for such initiatives, respondents pointed to mutual learning and personal rapport as decisive. Moreover, the resulting resilience-oriented projects connect directly to broader global development concerns such as climate adaptation, urban sustainability, and ecological governance, demonstrating that a subnational SP can serve as a platform for addressing issues that transcend the bilateral context.

#### Fostering inclusivity and cultural awareness: bridging societal divides

In addition to environmental objectives, the Manchester–Wuhan partnership targeted social cohesion, a goal that gained urgency during the COVID-19 pandemic. Instances of xenophobia and discrimination against Chinese nationals and students in Manchester—such as delivery drivers refusing to serve Chinese restaurants or racist remarks (BI2-b, PO2; PO5)—highlighted the partnership’s potential to promote cultural understanding. In response, local stakeholders established the Manchester China Friendship Programme (MCFP),^7^ a goal-oriented initiative conceived initially to counter anti-Asian prejudice. Over time, the MCFP evolved into a broader platform for academic collaboration, community events, and cultural exchanges (BI2-a).

According to senior management at the MCF, the programme’s success lay in its ability to engage students, community leaders, and volunteers around a shared narrative of inclusivity. “The current geopolitical climate and misunderstandings exacerbated by the COVID-19 pandemic highlight the need for change, especially among the younger generations,” one official noted (BI1-b). Participants confirmed that the MCFP significantly bridged cultural divides and strengthened inter-city ties. Recognising its impact, the University of Manchester co-funded the programme’s expansion, making it a stable fixture in the city’s engagement with Wuhan (BI2-b). This provides evidence of how non-material aspects, particularly relational trust and shared objectives, help a city-to-city partnership respond proactively to urgent societal challenges.

While it is plausible to interpret these cultural initiatives purely as efforts to enhance “soft power” or comply with certain policy incentives, local stakeholders insisted that personal-level connections had proven indispensable. Such networks, built up over decades, fostered the dialogue and resource-sharing needed to implement the MCFP rapidly and effectively. Indeed, the combination of relational trust and community-based collaboration enabled the partnership to address deeper social issues like racism and inter-group tensions—an outcome more difficult to achieve without this level of social capital in place.

The Manchester–Wuhan partnership’s commitment to tackling environmental and social challenges resonates with global challenges well beyond city-level ties. Initiatives such as Sponge City-inspired projects and the MCFP echo global development priorities, from sustainable urban planning to social inclusion. They also illustrate how subnational SPs can leverage shared objectives (e.g., climate resilience, cross-cultural solidarity) to produce outcomes that address urgent, internationally recognised goals—thereby aligning local objectives with broader IR and development discourses (Parnell, 2016; Curtis, 2021).

### A platform for sustained, long-term cooperation

The Manchester–Wuhan relationship demonstrates a long-term orientation across several key areas. In particular, two domains—higher education and evolving business collaborations—highlight how sister-city partnerships can build on formal agreements and develop varied forms of collaboration. Whereas the previous sections examined the partnership’s structural grounding and flexibility, here we focus on how it has generated a multi-decade trajectory of cooperation.

#### Enduring engagement in higher education

Higher education has consistently emerged as a central platform for the Manchester–Wuhan link. As a former MCC leader stated, "[I]t was understood from the beginning that achieving our goals in higher education might take years, possibly a decade, but the anticipated outcomes would justify the effort" (BG1-a). She further emphasised, "What we're seeing now... is the exchange of students, and universities working more closely together. It's no longer just about the council. We (i.e., MCC) set the foundation, but the result was that universities and higher education institutions began collaborating... you can see the investment from firms such as , which have significantly invested and conducted business in China" (BG1-c). This aligns with spatial synergies: higher‑education platforms operated as long‑horizon institutional anchors sustaining the partnership.

Programmes initiated by Alliance Manchester Business School (AMBS) at the University of Manchester (UoM) during the early 2000s exemplify this approach. As part of this, Wuhan municipal officials attended six-month governance training programmes, deepening ties (both formal and informal) between the cities (CG1). These exchanges enhanced subsequent collaborations as demonstrated by the AMBS becoming the first UK university licensed by China’s State Administration for Foreign Experts Affairs to train officials and executives from state-owned enterprises (SOEs), marking a significant milestone in the partnership (BI5-a).

This investment in education also foregrounds the spatial dimension of the sister-city agreement, leveraging Manchester’s position as a knowledge hub and Wuhan’s emphasis on governance reform. More recently, initiatives like the UK–China Infrastructure Academy, supported by the UK Government and led by AMBS, have drawn top business and government leaders from both cities, aligning infrastructure and educational goals (Manchester China Institute, Feb 2, 2018).^8^ By 2023, Manchester hosted over 8,000 students from mainland China, with Greater Manchester accommodating more than 10,000 (BI2-a).

#### Business collaboration: from formal introductions to long-term value

A further testament to the partnership’s longevity is found in business collaborations. Beginning in the early 2000s, Manchester City Council facilitated introductions for local firms—most notably ,^9^ Arena, and Mott MacDonald—to engage with infrastructure opportunities in Wuhan (BG1-c). , for example, took on pivotal roles in projects such as the Three Gorges Project, the Riverside Project, and the Wuhan Tianhe Airport expansion, transforming what began as a standard bidding process into a multi-year cooperative relationship.

A letter from ’s senior management to MCC in April 2004 noted Manchester’s crucial involvement in reinforcing Wuhan’s confidence in ’s proposals (internal doc 2 from MCC). Shortly thereafter, the then-mayor of Wuhan formally thanked Manchester for its endorsement, noting that the city’s support had been “instrumental” in ’s successful bid for the Tianhe Airport expansion and “reflected a commitment to shared development goals” (internal doc 1 from MCC). By emphasising trust and reciprocity rather than purely transactional motives, these exchanges propelled to establish a lasting foothold in China, ultimately enabling further achievements such as involvement in the design of Beijing’s Bird’s Nest stadium for the 2008 Olympics (BG1-c).

Crucially, these collaborations highlighted spatial synergies: Manchester’s expertise in urban planning and engineering meshed with Wuhan’s large-scale infrastructure ambitions, creating complementary advantages for both cities. Over time, personal and professional networks—cultivated through ongoing dialogue and project-specific engagements—helped each side invest in initiatives within the sister-city framework, thereby showcasing how subnational SPs can spur long-term, mutually beneficial development.

### Synthesis: non-material factors as the catalyst of subnational SPs

While formal agreements such as MoUs and institutional structures provide essential foundations for cooperation, these alone cannot fully account for the partnership’s resilience and adaptability. Instead, it is the interplay of formal agreements with non-material elements—trust, shared objectives, and place-specific synergies—that sustains and enhances this dynamic subnational SP. The handling of the Tiananmen Square backlash illustrates how Manchester navigated an ethical and political minefield without severing links with Wuhan, largely due to strong interpersonal rapport among local Chinese actors and city officials. Similarly, amid these political tensions between 2019 and 2021, the two cities pivoted towards hydrogen energy—a less contentious policy field that gained traction through the 2022 China–UK Hydrogen Energy Cooperation Forum (Palcan, 2022). This consistent reliance on interpersonal connections created a buffer against macro-political shocks, ensuring that cooperative efforts could continue in safer policy spaces. Spatial synergies are equally important: Manchester’s focus on higher education, creative industries, and green technologies meshes with Wuhan’s infrastructure-building drive and emphasis on knowledge transfer (Galaso, 2018; Mohan & Mohan, 2002). These complementarities support robust, multi-year strategies aligned with global agendas in climate resilience, inclusive growth, and sustainable urban design.

## Conclusion

This article reconceptualises city diplomacy as a form of SP, integrating institutional structures with non-material processes rooted in trust, shared objectives, and spatial synergies. By examining the Manchester–Wuhan sister-city agreement, we demonstrated how local actors can forge partnerships that transcend national policy fluctuations, adapt to emerging policy fields, and deliver tangible benefits for their communities. This approach suggests that neither exclusively state-centric models nor purely economic incentives can fully explain the dynamics of city diplomacy.

Theoretically, our findings extend the concept of SPs—traditionally centred on nation-states—by illustrating how subnational diplomacy can mobilise social capital in ways that circumvent or complement national-level directives. Rather than treating sister-city agreements as peripheral diplomatic tools, this framework highlights how municipal stakeholders build policy continuity, identify safe areas for cooperation (e.g., sustainable urban development), and cultivate interpersonal ties, thereby insulating partnerships from broader geopolitical disruptions.

From a development perspective, the Manchester–Wuhan experience aligns closely with global agendas such as the SDGs. Concrete initiatives like Sponge City-inspired environmental programmes and cross-cultural collaborations illustrate how subnational partnerships can effectively address societal goals more rapidly than national governments alone, emphasising municipal-level agency in co-producing developmental outcomes. These cases highlight the importance of municipal‑level agencies in co‑producing development outcomes, showcasing how trust‑based networks and bottom‑up innovation can expedite progress toward internationally recognised targets.

These insights also hold significant implications for policymakers. Municipal leaders should prioritise interpersonal exchanges, diaspora engagement, and local policy forums to strengthen networks and interactions foundational for lasting partnerships. National governments might consider granting local authorities’ greater autonomy or flexible frameworks, recognising that subnational actors can effectively pilot sensitive or innovative projects that national authorities may hesitate to undertake. Development agencies and NGOs could likewise partner with municipalities, thereby enhancing localised solutions in areas such as sustainability, education, and public health. An example of this research’s practical impact emerged during fieldwork when Duisburg—a German city maintaining a sister-city relationship with Wuhan—sought to learn from Manchester’s experiences. The authors facilitated an online discussion between the two municipal governments, illustrating the potential of knowledge-sharing across cities.

Future research might further investigate sister cities through comparative studies of diverse city partnerships—such as Liverpool–Shanghai, Milan–Shanghai, Duisburg–Wuhan, and Manchester–Wuhan—to test whether similar mechanisms operate across varying cultural and political contexts. Investigations into failing or suspended partnerships could also expose how the absence or erosion of non-material factors undermines long-term cooperation. Such inquiries promise deeper insights into municipal roles in international relations and development, offering valuable lessons for cities aiming to build global partnerships. Future comparative studies should particularly examine diasporic community involvement (Alejo, 2022) and employ comparative urbanism methodologies (Bunnell, 2018) to further test and generalise these critical findings. Systematic comparisons across different city pairs could analyse how variations in diaspora size, organisational capacity, and host-city receptivity shape partnership agendas and outcomes, clarifying when and where diaspora brokerage is most effective.

## Data Availability

No datasets were generated or analysed during the current study.

## Interview Questions for Stakeholders

### Common interview questions

1. Can you briefly introduce your role within your organisation?
2. How do you perceive the role of national and city diplomacy in international business and entrepreneurship?
3. What are the primary challenges for British companies investing in Wuhan or other Chinese cities, and conversely, for Chinese companies investing in Manchester or the UK?
4. To what extent do you think sister-city ties facilitate investment and economic partnerships? Please provide specific examples.
5. How has the evolving UK–China geopolitical relationship influenced local-level engagements, including your own activities?
6. How important is trust and understanding in successful international business relations, and how can diplomacy enhance these aspects?
7. Given the current state of UK–China relations, how do you foresee future investments between the two countries evolving?
8. Do you believe local networks and personal relationships can foster successful collaborations despite broader diplomatic tensions?

### Tailored interview questions (selected examples)

*For Manchester Government Officials*

- What led to the initial selection of Wuhan for a sister-city partnership with Manchester?
- Could you highlight key milestones in the development of Manchester–Wuhan relations over the past two decades?
- How has Manchester City Council responded to geopolitical shifts, such as the recent cooling of UK–China relations?

*For Investors and Business Representatives*

- Why did you select Wuhan or other Chinese cities for investment, and how have city-diplomacy efforts influenced your decisions?
- How do you manage the balance between national-level geopolitical issues and local business opportunities?

*For Chinese Community Leaders*

- How has the local Chinese community influenced or facilitated business and cultural exchanges between Manchester and Chinese cities?
- Could you give examples of specific community initiatives that have enhanced Manchester–Wuhan relations?

## Footnotes

1 The concept of social capital has been defined in various ways (see Bourdieu, 1986; Coleman, 1990; Putnam, 1993; Mohan and Mohan, 2002). While it broadly encompasses features of social organisation (e.g., trust, norms, and networks) that facilitate cooperation for mutual benefit, we do not seek to offer a comprehensive conceptualisation here. Instead, we focus on how certain intangible, people-centred dimensions—such as trust, shared objectives, and reciprocity—operate as non-material factors in city-level partnerships, drawing on key insights from the social capital literature to underscore the role of these ‘soft’ variables in subnational diplomacy.

2 We list each city pair using the order employed in the respective official agreement; in comparative lists of multiple pairs, we default to alphabetical order by the first city.

3 The term “Chinese community” in this paper refers to a diverse collective of individuals and groups connected through a shared Chinese heritage, either by self-identification or external categorisation. This community includes individuals from mainland China, Hong Kong, Taiwan, and Southeast Asian countries such as Vietnam and Singapore, as well as the Chinese diaspora in the West Indies. Additionally, it encompasses British-born Chinese individuals who contribute to the dynamic and evolving nature of this identity. Drawing on Benedict Anderson’s (1991) concept of imagined communities, the notion of a unified Chinese identity is largely constructed through shared cultural practices, experiences, and heritage, despite the diversity of migration histories, socioeconomic statuses, and generational divides within the group (Anderson, 1991).

4 “Uncle Charlie”, as described by the current Chinese community leader in Manchester (BI6-d), played a significant role in establishing links between the local Chinese diaspora and city authorities, contributing directly to the formation of the Manchester–Wuhan partnership (BI6-d).

5 See https://ukncc.org/ for more details.

6 The sponge city concept refers to “a way of urban management that allows cities to naturally absorb, store and purify rainwater to resolve waterlogging issues, prevent urban flooding, improve water storage and discharge capacity, and enhance water quality” (The River Trust, 2023).

7 This programme is in collaboration with the MCI and the UoM International Office, established the MCFP in 2021 (see Manchester China Friendship Programme - Manchester China Institute 曼彻斯特中国研究院 - The University of Manchester for more details).

8 Online available at: Manchester China Institute 曼彻斯特中国研究院 | The University of Manchester.

9 . Online available at: https://www..com/contact-us/mainland-china/.
